# Advanced neuroimaging techniques in epilepsy

**DOI:** 10.1097/WCO.0000000000001007

**Published:** 2022-04-01

**Authors:** John S Duncan, Karin Trimmel

**Affiliations:** 1 Department of Clinical and Experimental Epilepsy UCL Queen Square Institute of Neurology London WC1N 3BG, UK; 2 MRI Unit Chalfont Centre for Epilepsy. Chalfont St Peer SL9 0LR, UK; 3 Department of Neurology Medical University of Vienna A-1090 Vienna, Austria

**Keywords:** epilepsy, MRI, functional MRI, PET

## Abstract

**Purpose of review:**

We review significant advances in epilepsy imaging in recent years.

**Recent findings:**

Structural MRI at 7T with optimization of acquisition and post-acquisition image processing increases the diagnostic yield, but artefactual findings remain a challenge. MRI analysis from multiple sites indicates different atrophy patterns and white matter diffusion abnormalities in temporal lobe and generalized epilepsies, with greater abnormalities close to the presumed seizure source. Structural and functional connectivity relate to seizure spread and generalization; longitudinal studies are needed to clarify the causal relationship of these associations. Diffusion MRI may help predict surgical outcome and network abnormalities extending beyond the epileptogenic zone. 3-dimensional multimodal imaging can increase the precision of epilepsy surgery, improve seizure outcome and reduce complications. Language and memory fMRI are useful predictors of postoperative deficits, and lead to risk minimization. FDG PET is useful for clinical studies and specific ligands probe the pathophysiology of neurochemical fluxes and receptor abnormalities.

**Summary:**

Improved structural MRI increases detection of abnormalities that may underlie epilepsy. Diffusion, structural and functional MRI indicate the widespread associations of epilepsy syndromes. These can assist stratification of surgical outcome and minimize risk. PET has continued utility clinically and for research into the pathophysiology of epilepsies.

## Introduction

The advent of magnetic resonance imaging (MRI) in the 1980s has led to a growth of epilepsy surgery and the increasing sensitivity of MRI is revealing more and more subtle underlying structural lesions. Functional MRI (fMRI) in epilepsy is used both to identify the epileptogenic zone as well as essential brain regions. Language and memory fMRI are increasingly applied to map eloquent cortex and predict the extent of cognitive deficits following epilepsy surgery. Simultaneous EEG-fMRI, MEG, PET and SPECT are used to investigate the epileptogenic focus. Furthermore, functional connectivity studies have improved our understanding of disease-specific effects on brain networks.Diffusion MRI (dMRI) and its derivatives of network analysis and tractography can identify the structural basis of connectivity and, in individual patients, guide optimal resections that mitigate the risk of causing deficits. Positron emission tomography (PET) antedates MRI and mapping cerebral glucose uptake can be a useful method to lateralize and broadly localize the epileptogenic zone. Ligand PET has a unique ability to probe and visualize the neurochemical abnormalities that underpin epilepsy and seizures. The field is moving quickly, in this review we consider the principal highlights of research in 2020 and 2021.

In this review, we consider the impact of recent advances in MRI, imaging processing and diffusion imaging on presurgical evaluation, followed by the use of language and memory fMRI to mitigate the adverse effects of epilepsy surgery. We then consider methods to help localize the epileptogenic zone, followed by studies of epileptic networks and the use of PET to probe the neurochemical bases of epilepsies. We conclude with a consideration of multimodal integration of imaging data.

### Magnetic resonance imaging

Several papers have noted the increased yield of visualizing subtle structural abnormalities that were not evident on 1.5T and 3T acquisitions, particularly subtle malformations such as focal cortical dysplasia and polymicrogyria, leading to taskforce recommendations on the use of 7T acquisitions [1]. There is the inevitable caveat that not every structural abnormality is causally related to epilepsy [2]. In a systematic review of 275 patients in 16 studies, the overall extra yield was 31%, with particular yield of focal cortical dysplasia, hippocampal sclerosis and gliosis [3**]. In general, T2* sequences gave good yield. 3D FLAIR was best for detection of focal cortical dysplasia and 3D T2* usefully clarified the internal structure and extent of dysplasia. T2 Turbo spin echo and susceptibility weighted images were best for imaging the subfields of the hippocampus. Susceptibility weighted images were particularly good for identifying vascular abnormalities. A further systematic review gave comparable results [4]. In individuals with lesions seen at 3T, 7T imaging was associated with increased conspicuity and definition of boundaries, but with increased artefacts, particularly in the temporal lobes [5], and there was benefit from post-acquisition morphometric analysis in individuals with unremarkable scans at 3T [6].

Susceptibility weighted imaging at 7T can demonstrate the anatomy of blood vessels with high definition, with the potential to highlight subtle abnormalities of the neocortex and hippocampi [7*].

In a preliminary study, volumetric Glutamate Chemical Exchange Saturation Transfer (GluCEST) imaging at 7T showed increased levels of glutamate in hippocampi and subiculum, ipsilateral to seizure onset in temporal lobe epilepsy (TLE) with no structural lesions [8*]. This technique shows promise to understand abnormalities of glutamate levels in cerebral networks, particularly if whole brain data can be acquired. Magnetic resonance spectroscopy at 7T offers better differentiation of individual peaks attributed to glutamate, γ-aminobutyric acid (GABA) and glutathione and has been applied in 8cm^3^ single voxels in individuals with epilepsy [9]. Going forward, multivoxel spectroscopic imaging of large brain areas hold promise to investigate neurochemical changes in cerebral networks.

### Image processing

Morphometric analysis has been shown to aid detection of subtle focal cortical dysplasia. Using an MP2RAGE2 sequence that improves homogeneity of T1-contrast, and corrects B_1_ inhomogeneities, junction images from morphometric analysis show abnormalities with more sensitivity and precision [10*]. The Epilepsy ENIGMA consortium is a multisite enterprise that pools imaging and clinical data from thousands of patients and controls in homogenous formats, enabling studies with adequate power to provide definitive conclusions. Support vector machines, that are supervised learning models with associated learning algorithms, and deep learning approaches, that are based on **multi-layered neural networks**, have been used to analyse brain MRI from TLE patients with and without hippocampal sclerosis, and showed superiority of the support vector machine approach for diagnostic accuracy [11]. In a cross-sectional analysis, TLE patients showed atrophy in connected temporo-limbic cortical hubs, in contrast to those with genetic generalized epilepsy, who had predilection to fronto-central atrophy [12*].

### Diffusion MRI

A multicentre ENIGMA study of diffusion MRI parameters in TLE and genetic generalized epilepsy found that fractional anisotropy (FA) in white matter was widely reduced, particularly in corpus callosum, cingulum and external capsule, and associated with smaller increases in mean diffusivity. In TLE with hippocampal sclerosis (HS) these changes were particularly seen in the ipsilateral parahippocampal cingulum and external capsule. Greater changes were associated with earlier onset and longer duration of epilepsy, suggesting that the effects may be a consequence of epilepsy [13*]. Longitudinal studies are needed to tease out the causal relationship of these changes.

In generalized epilepsy, similar changes were seen in both hemispheres. Interestingly, FA alterations were more marked in refractory generalized epilepsy and increased nodal volume in those with generalized epilepsy that was controlled with medication [14]. At this time, the causal relationship of drug responsiveness and diffusion changes is not clear. Abnormalities of structural and functional connectivity were shown to be more marked closer to the presumed seizure onset zone, consistent with the findings of the ENIGMA study [15]. A consistent finding was that functional connectivity parameters were more abnormal in those with TLE and HS than those without HS [16].

Diffusion MRI may also correlate with patterns of seizure propagation. In a correlation of tract diffusion parameters and SEEG, seizure spread was associated with greater FA in relevant tracts, suggesting an augmentation of structural connectivity [17*]. In contrast, TLE patients with focal to bilateral tonic-clonic seizures (FBTCS) had greater network abnormalities across the brain, than those without FBTCS, suggesting that abnormal connectivity may underlie the tendency to seizure generalization [18].

In presurgical evaluation, reduced mean Kurtosis derived from diffusion imaging has been suggested as a useful adjunct to delineating the epileptogenic zone [19*]. A further interesting possible imaging tool to localizing the epileptogenic zone comes from an experimental study of mesial TLE with combined measures of T1, diffusion, blood volume and blood brain barrier integrity at 9.4T [20**]. Applying these imaging methods in vivo in human subjects will be of great interest.

In epilepsy surgery, the visualization of the optic radiation with neuronavigation during surgery reduces the risk of causing damage and visual field deficits [21*]. The same principles may be applied to other tracts, e.g. sustaining motor and language functions, to minimize risk of post-operative deficits. Diffusion imaging data may also be useful for predicting the outcome of surgery. Patients with TLE having surgical resections, who had abnormal integration of structural network nodes were less likely to become seizure free after surgery, findings that are concordant with previous studies of network abnormalities as a prognostic factor [22]. Patients with more abnormal nodes, not resected by surgery, were more likely to continue to have seizures [23*] suggesting that this may be a useful preoperative marker of prognosis after surgery.

In individual patients having laser interstitial thermal therapy (LITT) for HS, seizure freedom was more likely to occur if ablated hippocampal tissue had higher apparent diffusion coefficient, suggesting that this parameter may aid optimal LITT targeting in the mesial temporal lobe [24]. Memory impairment is a significant concern after temporal lobe epilepsy surgery. Analysis of structural connectomes and tracts were a stronger predictor of post-operative memory decline than were hippocampal volume and clinical data [25]. Combining these data with results of memory fMRI may be a useful way to give reliable individual prognosis, and possibly lead to mitigation strategies.**Language fMRI**

The implementation of fMRI tasks that primarily activate the brain areas that are to be removed in surgery may improve the prediction of postoperative naming decline. In TLE patients, auditory and visual naming reliably activate temporal lobe regions [26], and stronger preoperative recruitment of language regions contralateral to the seizure onset relates to better postsurgical language outcome in TLE [27*]. An active area of research is how the analysis of white matter tracts can aid surgical planning and minimize language decline after surgery. Language laterality also reflects in differences of underlying language-specific white matter tracts [28], which could thus be integrated in longitudinal studies focussing on language outcome after epilepsy surgery..

Interpretation of language fMRI results may be challenging in pediatric epilepsy patients, who tend to show more bilateral language representation owing to more extensive lesions as well as ongoing neuroanatomical development. Concordance rates of intracarotid amobarbital procedure (IAP) and language fMRI continue to support the use of fMRI as a replacement for IAP [29*], yet the optimal choice of fMRI task paradigms in this patient group is still of debate. Contrary to task-based fMRI, resting-state fMRI (rs-fMRI) may also be acquired in very young patients, or in people who are cognitively impaired. Hemispheric dominance derived from rs-fMRI shows good concordance with task-based fMRI in both adult and pediatric epilepsy patients [30*,^31^*]. An important extension of these studies would be to explore whether rs-fMRI can be used to accurately predict language outcome following epilepsy surgery.

### Memory fMRI

A recent meta-analysis addressed the concordance of memory fMRI and IAP for memory lateralization in TLE, concluding that concordance is lower than expected (46.8%), and varying widely across studies. Concordance was found to be relatively low in patients who were identified as bilateral on IAP and in patients with structural lesions like HS. The specificity of IAP to predict postoperative memory decline is highly debated, particularly regarding non-verbal memory, and it has been suggested that fMRI is superior to IAP in the presurgical assessment of memory lateralization [32*]. Including fMRI paradigms of associative memory, encoding, and recall (as compared to recognition), as well as using overt responses to monitor task performance may improve sensitivity and specificity when imaging memory function in TLE [33*], which will ultimately improve the prediction of postoperative memory deficits.

Future studies should address language and memory outcomes in patients receiving different types of treatments, including laser ablation, and include patients with extratemporal lesions. Replication studies across multiple centres are warranted to define clinical standards for different patient groups.

### Assessment of seizure onset zone: ESI, MSI, EEG-fMRI

Electrical source imaging (ESI) based on scalp EEG combined with MRI data has been demonstrated to localize interictal spikes [34]. ESI of fast oscillations (>40Hz) using high-density (256-channel) EEG was shown to more accurately localize the epileptogenic zone compared to conventional EEG approaches with reduced electrode coverage [35*]. ESI was confirmed to have added diagnostic value to routine presurgical assessment and may change management plans in up to one third of focal epilepsy patients, with the main indication of planning of intracranial recordings [36*]. This supports earlier findings from magnetoencephalography source imaging (MSI), which is less widely available due to higher costs.

Simultaneous scalp EEG-fMRI can assist identification of the epileptogenic focus in pharmacoresistant focal epilepsy by mapping haemodynamic changes associated with interictal epileptic discharges [37*]. Ictal EEG-fMRI may be obtained in patients with frequent seizures. Good seizure outcome has been reported in cases where surgery or placing of intracranial electrodes was determined largely by localization from the scalp EEG-fMRI result [38*]. Intracranial EEG-fMRI improves the spatial discrimination of IED generating tissue, and recent findings suggest that instead of using the maximum BOLD response alone, taking into account all available clinical information in the assignment of the most clinically relevant BOLD cluster better identifies IED origin [39*]. The combination of EEG-fMRI with two additional functional imaging techniques (high-resolution scalp EEG, MEG, or PET) is superior to individual modality accuracy in the identification of the epileptogenic zone. Combined techniques show up to 80% accuracy rates compared to SEEG, particularly in patients with multilobar seizure onset [40*].

### Network studies

Focal epilepsy is increasingly recognized to be a network disorder, with broad functional and structural network abnormalities that extend far beyond the epileptogenic zone. Functional connectivity analyses such as psychophysiological interaction, dynamic causal modelling, or graph theory metrics are increasingly applied and allow the study of these networks. In TLE patients, graph theory and causal inference analyses based on rs-fMRI connectivity suggest that regionally increased connectivity in the affected hippocampus and amygdala, and decreased connectivity in the lateral temporal lobe relates to high seizure frequency. The extent of neuropsychological impairment, however, seems to be associated with a more global disorganization of the connectome [41*].

Thalamo-cortical connectivity has long been recognized as a contributor to the pathogenesis of epileptic activity and seizure propagation. In adult TLE patients, task-related thalamic functional connectivity profiles have recently been suggested as imaging biomarkers of secondary generalization of focal seizures [42*]. In pediatric patients, hyperconnectivity of the anterior nucleus of the thalamus ipsilateral to the epileptogenic zone was observed [43], and ongoing research investigates whether connectivity measures are predictive of treatment response to deep brain stimulation (DBS).

Future studies will evaluate how both regional and global network metrics can be integrated into presurgical evaluation, prediction of postsurgical seizure and cognitive outcome, drug treatment response, and tailored treatment approaches, particularly with integration of measures of structural and functional connectivity.

### Positron Emission tomography

PET with ^18^F-FDG has been used for over 40 years to help identify areas of focal cerebral hypometabolism, with a yield in TLE of about 60% and 30% in extra temporal lobe epilepsies. Dynamic PET acquisition with a quantitative assessment, rather than static PET imaging, has been suggested to have increased sensitivity to identify areas of hypometabolism when conventional FDG-PET was unremarkable, and merits further evaluation [44].

Sophisticating the stratification of risk of SUDEP is an ongoing issue, and recent PET studies are providing useful data on this. In individuals with drug-refractory focal epilepsy, bilateral frontal hypometabolism, particularly of medial and inferior frontal cortex, has been reported to be associated with SUDEP and other factors associated with a high risk for SUDEP [45] and merits further enquiry. In a series of patients with temporal, or temporal “plus” epilepsy investigated with FDG-PET and video-EEG telemetry, ictal asystole was associated with right posterior insula hypometabolism [46].

Postictal generalized suppression (PGES) may be associated with an increased risk of SUDEP. In a study from the same centre, compared to those without PGES, individuals with this EEG feature had more hypometabolism in right temporal pole, right parahippocampal gyrus and a predominantly right sided network of temporal lobe, and connecting cortical and subcortical structures [47].

Whilst FDG-PET is used for clinical epilepsy studies, PET with specific ligands is useful in research endeavours to probe the neurochemistry associated with epileptogenesis and the pathophysiology of epilepsy and seizures.

PET with translocator protein (TSPO) showed increased TSPO corresponding to neuroinflammation in rodent TLE models, and an association with microglial activation, astrogliosis and cell death. Clinical studies with[^11^C]- PBR28 in TLE found increased TSPO ipsilateral to seizure foci. Further studies are needed to evaluate the clinical role of this ligand and there are technical challenges [48].

Synaptic vesicle glycoprotein 2A (SV2A) binding has been shown to be reduced in brain removed in epilepsy surgery. In vivo binding of the SV2A PET tracer [(11) C]UCB-J was reduced in hippocampal sclerosis, with the reduction being greater than that accounted for by hippocampal atrophy and by reduction in FDG uptake, suggesting a further molecular process [49].

A novel PET tracer, derived from 4-[2-(phenylsulfonylamino)ethylthio]-2,6-difluoro-phenoxyacetamide labeled with 11C ([11C]K-2), was recently developed that specifically binds to AMPA receptors in vivo. Increased binding was found in the mesial temporal lobe of patients with mesial temporal lobe epilepsy, which correlated with the AMPA receptor protein distribution in resection specimens [50]. Given the role of AMPA receptors in the pathophysiology of epilepsy and a range of other conditions, this tracer promises to be of great importance.

### Integrated multimodal imaging

Multimodal neuroimaging, including MRI, with derivatives of visualization of arteries and veins and white matter tracts PET, SPECT, MEG and EEG data, has been shown to be useful in the planning of intracranial EEG and resections for refractory focal epilepsy. The translation of these methods has been demonstrated in a cohort of 467 patients over 4 years, 351 of whom underwent surgery, with 50% seizure free at one year [51].

## Conclusion

In individuals with drug-refractory focal epilepsy, MRI acquisition at 7T and post-acquisition processing increase the detection of subtle abnormalities that are important in the consideration of surgical treatment. The finding of widespread diffusion abnormalities is an adverse prognostic sign. Integration of structural MRI, diffusion MRI tractography and fMRI enables neuronavigation in surgery with the potential to optimize outcome and minimize risk of causing deficit. Prospective studies are needed to determine the benefits. Group studies demonstrate the abnormalities of structure, diffusion and functional connectivity associated with epilepsy syndromes, and associations of seizure generalization. Longitudinal studies are needed to determine the causal relationship of these changes. Ligand PET is a highly specialised technique that is not widely available but has the potential to clarify the pathophysiology of epilepsy and identify novel treatment targets. The potential clinical relevance of the methods described are summarised in Table 1

## Figures and Tables

**Table 1:** The potential clinical relevance of imaging methods

Magnetic resonance >Imaging	7 Tesla acquisition	Identification of occult lesions that are relevant for presurgical evaluation
	Glutamate Chemical Exchange Saturation Transfer	Identify elevated levels of glutamate
Image processing		Improved visualisation of subtle abnormalities
Diffusion imaging		Visualisation of critical nerve fibre tracts to enable surgical navigation to avert damage
Language and memory fMRI		Prediction and mitigation of risk of impairment following epilepsy surgery
Electrical and magnetic source imaging EEG-fMRI		Non-invasively infer localisation of irritative zone
Positron emission tomography		Ligand PET has the potential to better localize the epileptogenic zone
Integrated multimodal imaging		Visualize normal and abnormal structure and function in common anatomical space, enabling sophisticated surgical planning.

## Key points

Include 3-5 key bullet points that summarise your article after the main text. Each bullet is to be no longer than one sentence.

- Structural MRI at 7T, optimized acquisition and post-processing increase the detections of covert lesions underlying focal epilepsies.
- There are widespread abnormalities of white and grey matter, with syndrome specific features and greater abnormalities close to the presumed epileptogenic zone.
- Multimodal 3D neuronavigation and fMRI may improve the outcome of epilepsy surgery
- Ligand PET can identify neurochemical and receptor abnormalities involved in epileptogenesis
